# GPR55 Antagonist CID16020046 Attenuates Obesity-Induced Airway Inflammation by Suppressing Chronic Low-Grade Inflammation in the Lungs

**DOI:** 10.3390/ijms25137358

**Published:** 2024-07-04

**Authors:** So-Eun Son, Ye-Ji Lee, Yoon-Jung Shin, Dong-Hyun Kim, Dong-Soon Im

**Affiliations:** 1 Department of Biomedical and Pharmaceutical Sciences, Graduate School, Kyung Hee University, Seoul 02447, Republic of Korea; seson@khu.ac.kr (S.-E.S.); youngsimm@naver.com (Y.-J.L.); nayo971111@naver.com (Y.-J.S.); 2 Department of Fundamental Pharmaceutical Sciences, Graduate School, Kyung Hee University, Seoul 02447, Republic of Korea; dhkim@khu.ac.kr

**Keywords:** GPR55, lysophosphatidylinositol, obese, inflammation, lung, CID16020046

## Abstract

GPR55 is a receptor for lysophosphatidylinositols (LPIs) in digestive metabolites. Overnutrition leads to obesity, insulin resistance, and increased LPI levels in the plasma. The involvement of LPIs and GPR55 in adiposity, hepatic steatosis, and atherosclerosis has been previously elucidated. However, the therapeutic efficacy of GPR55 antagonists against obesity-induced airway inflammation has not been studied. The present study investigated whether CID16020046, a selective antagonist of GPR55, could modulate obesity-induced airway inflammation caused by a high-fat diet (HFD) in C57BL/6 mice. Administration of CID16020046 (1 mg/kg) inhibits HFD-induced adiposity and glucose intolerance. Analysis of immune cells in BALF showed that CID16020046 inhibited HFD-induced increase in immune cell infiltration. Histological analysis revealed the HFD induced hypersecretion of mucus and extensive fibrosis in the lungs. CID16020046 inhibited these HFD-induced pathological features. qRT-PCR revealed the HFD-induced increase in the expression of *Ifn-γ*, *Tnf-α*, *Il-6*, *Il-13*, *Il-17A*, *Il-1β*, *Nlrp3*, and *Mpo* mRNAs in the lungs. CID16020046 inhibited the HFD-induced increases in these genes. The expression levels of adipokines were regulated by the HFD and CID16020046. *AdipoQ* in the lungs and gonadal white adipose tissue was decreased by the HFD and reversed by CID16020046. In contrast, *Lep* was increased by the HFD and suppressed by CID16020046. The findings suggest the potential application of the GPR55 antagonist CID16020046 in obesity-induced airway inflammation.

## 1. Introduction

G protein-coupled receptor 55 (GPR55) is a receptor for lysophosphatidylinositols (LPIs) and other digestive metabolites including oleoylethanolamide and lysophosphatidylcholines [1,2,3,4,5,6,7,8]. *GPR55* mRNA has been described in the human white adipose tissue and liver [9,10]. In patients with obesity, GPR55 expression is increased in visceral adipose tissue [10]. GPR55 expression is observed in pancreatic β-cells in rodents, but not in α- and δ-cells [3,9]. The levels of GPR55 are higher in the jejunum and ileum than in the colon and stomach of mice [7]. Expression of GPR55 is also observed in some brain regions, placenta, adrenal, proximal tubule cells of kidney, spleen, lung, and immune cells such as mast cells, monocytes, and natural killer cells in humans [11,12,13,14]. Kurano et al. described that albumin-bound LPIs induce the secretion of inflammatory cytokines by macrophages via the GPR55 and p38 mitogen-activated protein kinase (MAPK) pathways [15]. GPR55 couples to Gα_12/13_ or Gα_i/o_ in distinct cell types [16,17]. The GPR55 agonist O-1602 [18,19] induces increased concentration of intracellular calcium ion (Ca^2+^) in isolated pancreatic β cells, 3T3-L1 adipocytes, and Hep3B hepatoma cells [20,21,22].

LPI levels are increased in patients with obesity and are higher in patients with nonalcoholic steatohepatitis than in patients with only steatosis [10,23]. Plasma levels of LPIs are higher in db/db mice than in db/m mice [15]. Previously, we reported that the levels of LPIs are increased in the liver in mice fed a high-fat diet (HFD) and that the administration of O-1602 induces significant hepatic steatosis in the mice [22]. HFD-induced hepatic steatosis is reversed by the administration of CID16020046, a specific GPR55 antagonist [22,24]. Activation of GPR55 by LPIs promotes adipocyte differentiation and induces the expression of lipogenic genes in visceral and subcutaneous adipose tissues [10]. Increased LPI concentrations have been directly correlated with pro-inflammatory cytokine concentrations in the blood of patients with obesity [10]. Therefore, the activation of GPR55 by increased LPIs in HFD-fed mice results in the accumulation of fat in hepatocytes and adipocytes, and may be associated with human obesity and metabolic disorders. A genome-wide association study identified a variant of *GPR55* that displayed a phenotype of type 2 diabetes and coronary artery calcification [25]. Furthermore, CID16020046 blockade of GPR55 reportedly improved lipid profiles and protected against atherosclerosis progression in mice fed a HFD [26], suggesting the therapeutic potential of GPR55 in obesity-related diseases [27].

Overweightness or obesity has been associated with an increased prevalence of asthma symptoms [28]. Patients that are overweight have more severe asthma than those with normal weights, and clinical severity of asthma is positively associated with body mass index [29,30]. Chronic low-grade systemic inflammation has been speculated to be a mechanism in obese individuals; high levels of pro-inflammatory molecules released from adipose tissue into the systemic circulation could contribute to airway inflammation in patients with obesity [31,32]. In the present study, we aimed to investigate whether GPR55 antagonists can regulate obesity-induced airway inflammation by administering CID16020046 to C57BL/6 mice fed a HFD for 15 weeks.

## 2. Results

### 2.1. CID16020046 Reverses HFD-Induced Glucose Intolerance

**Figure 1:**
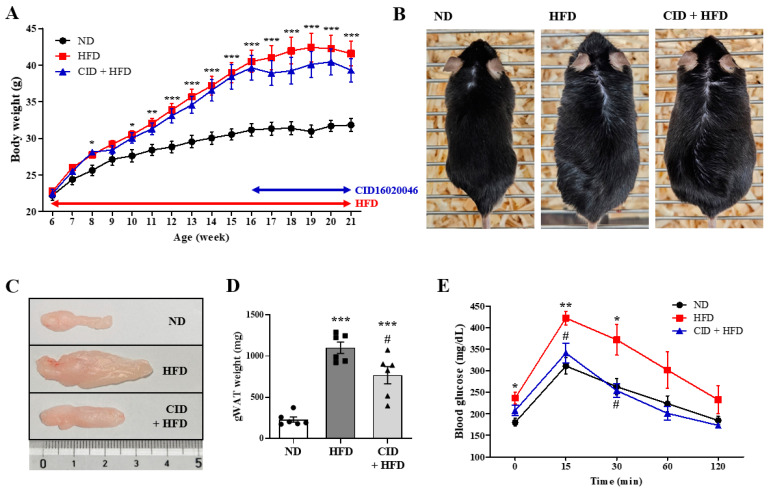
CID16020046 inhibits high-fat diet (HFD)-induced adiposity and glucose intolerance. Effects of CID16020046 on HFD-induced weight gain, glucose intolerance, and adiposity were measured in C57BL/6 mice. (**A**) Changes of body weights. Mice were fed the HFD from 6 weeks of age for 15 weeks, and administered CID16020046 intraperitoneally at the dose of 1 mg/kg from weeks 10 through 15. (**B**) Representative photos of 21-week-old mice. (**C**) Photos of gonadal white adipose tissues were measured. (**D**) Histogram of weights of adipose tissue. (**E**) Oral glucose tolerance test results at 21-weeks-of-age. Values are presented as the mean ± SEM of six mice per group. * *p* < 0.05, ** *p* < 0.01, and *** *p* < 0.001 vs. ND group, # *p* < 0.05 vs. HFD group.

Based on previous studies of obesity, adiposity, and hepatic steatosis, an HFD-induced obesity model was used in C57BL/6 mice. Male C57BL/6 mice were fed the ND or HFD for 15 weeks beginning at 6 weeks of age (Figure 1A). The HFD plus CID16020046 group was fed the HFD for 15 weeks and from weeks 10–15 treated five times each week with intraperitoneal injections of CID16020046 (1 mg/kg) for the last 5 weeks (Figure 1A). The HFD induced an increase in body weight by 30.67% compared to the ND group, whereas CID16020046 treatment showed a tendency to inhibit weight gain, although the effect was not significant (Figure 1A,B). The HFD increased the weights of the gonadal white adipose tissue by 379.7% (Figure 1C,D). CID16020046 administration significantly suppressed this increase (Figure 1C,D). To confirm obesity-induced glucose intolerance, OGTT was performed on the last day of week 21. Blood glucose levels were higher in the HFD-fed mice than in ND mice. CID16020046 administration improved normal glucose tolerance in mice (Figure 1E). Therefore, GPR55 inhibition improved HFD-induced glucose intolerance. 

### 2.2. CID16020046 Suppresses AHR and Immune Cell Infiltration in the BALF

**Figure 2:**
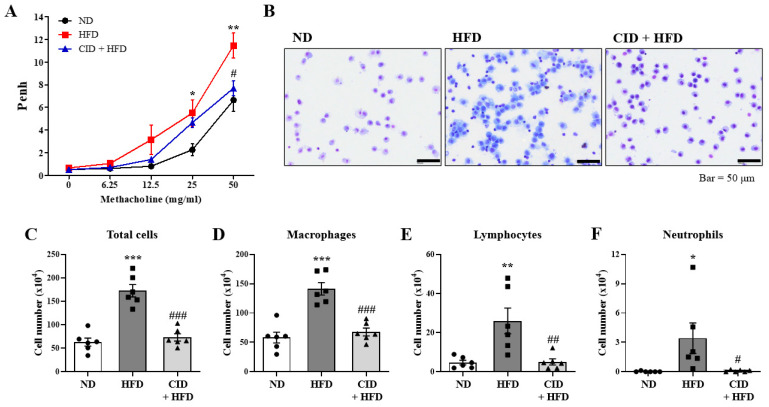
CID16020046 inhibits high-fat diet (HFD)-induced AHR and immune cell infiltration in bronchoalveolar lavage fluid (BALF). (**A**) Effects of the HFD and CID16020046 on AHR measured using noninvasive whole-body plethysmography. (**B**) Cells in BALF were stained using May–Grünwald stain and counted. (**C**–**F**) Enumeration of total cell counts (**C**), macrophages (**D**), lymphocytes (**E**), and neutrophils (**F**) in BALF. The results are presented as the mean ± SEM (*n* = 6). * *p* < 0.05, ** *p* < 0.01, and *** *p* < 0.001 vs. the ND group, # *p* < 0.05, ## *p* < 0.01, and ### *p* < 0.001, vs. the HFD-treated group.

To evaluate obesity-induced asthma, Penh was used to determine the effect of CID16020046 on AHR. Obese mice exhibited significant increases in Penh values compared to control mice at methacholine doses of 25 and 50 mg/mL, implying obesity-associated asthma (Figure 2A). CID16020046 treatment suppressed the increase in Penh at a methacholine dose of 50 mg/mL (Figure 2A).

To assess obese-induced airway inflammation, we analyzed immune cells in BALF. As shown in Figure 2B, total cell numbers were significantly increased by a HFD, while CID16020046 treatment significantly inhibited this increase (Figure 2B,C). The predominant immune cells were macrophages and lymphocytes (Figure 2D,E), with fewer neutrophils (Figure 2F), indicating that obesity-induced airway inflammation is a distinct type from allergic airway inflammation caused by eosinophils [33,34]. CID16020046 treatment significantly inhibited the HFD-induced increase in the numbers of macrophages, lymphocytes, and neutrophils (Figure 2C–F). We were not able to observe eosinophils in the BALF.

### 2.3. CID16020046 Suppressed Pathological Changes in the Lungs

**Figure 3:**
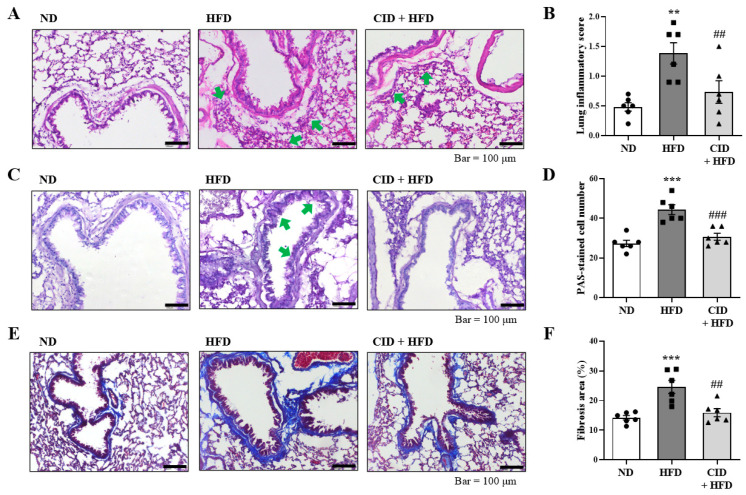
CID16020046 inhibits high-fat diet (HFD)-induced histologic responses in the lungs. (**A**) Panels show hematoxylin and eosin-stained sections of lung tissues from the ND group, HFD group, and CID16020046 (1 mg/kg)-treated HFD group. Small navy-blue dots around the bronchioles are neutrophils. Neutrophils were rarely observed in the ND group, whereas they accumulated densely around the bronchioles in the HFD group (green arrows). (**B**) Histogram of lung inflammation. (**C**) Panels show PAS-stained sections of lung tissues from the ND group, HFD group, and CID16020046 (1 mg/kg)-treated HFD group. In PAS staining, mucin is stained purple. In the HFD group, a darker and thicker purple color is observed surrounding the bronchiole compared to the ND group (green arrows). (**D**) Histogram of PAS-stained cells. (**E**) Panels show Masson’s trichrome-stained sections of lung tissues from the ND group, HFD group, and CID16020046 (1 mg/kg)-treated HFD group. (**F**) Histogram of the area of fibrosis. The results are presented as the mean ± SEM (*n* = 6). ** *p* < 0.01 and *** *p* < 0.001 vs. the ND group, ## *p* < 0.01, and ### *p* < 0.001, vs. the HFD-treated group.

Obesity-induced airway inflammation was determined using histological analysis. In H&E-stained sections from mice, the HFD induced immune cell accumulation around the bronchial airways, whereas CID16020046 significantly suppressed this increase (Figure 3A,B). As shown in Figure 3C, the HFD induced mucus hypersecretion in the airways, and PAS-positive goblet cells were observed around the bronchial airways (Figure 3C,D). The administration of CID16020046 inhibited this hypersecretion (Figure 3C,D). In MT-stained sections, the HFD induced a significant increase in blue-stained fibrotic areas, whereas CID16020046 suppressed the areas of fibrosis (Figure 3E,F).

### 2.4. CID16020046 Suppresses M1 Macrophages in the Lungs

**Figure 4:**
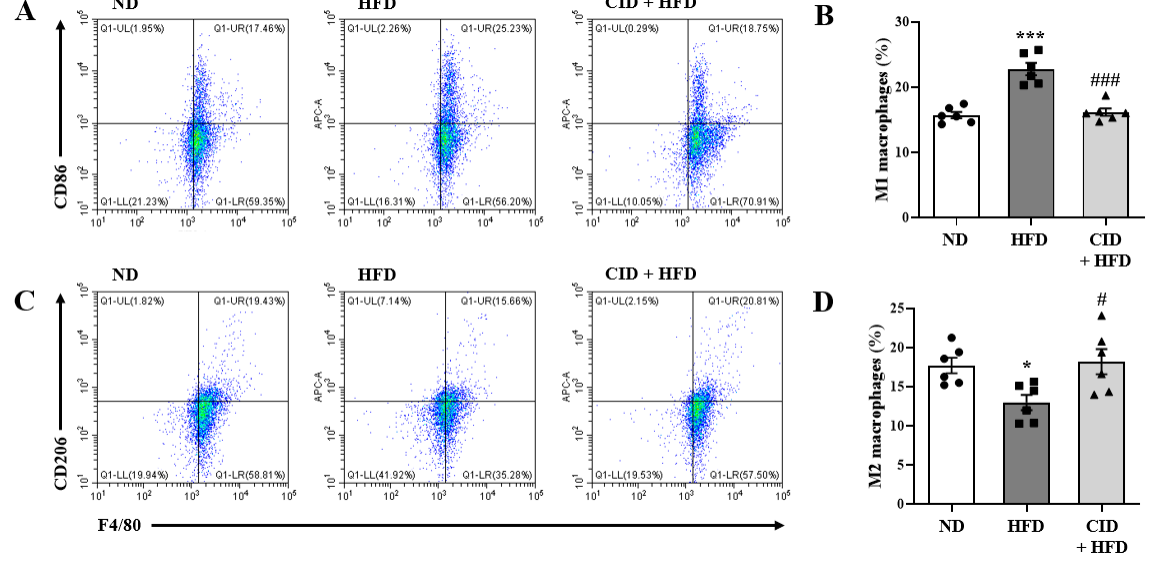
CID16020046 inhibits high-fat diet (HFD)-induced increase in M1/M2 macrophages in the lungs. For the identification of M1 and M2 macrophages from mouse lung tissue, single-cell suspensions were prepared using collagenase type II. Cells were first gated for singlets based on FSC and SSC parameters. F4/80-positive cells were included from the analysis. From these F4/80-positive cells, M1 (CD86^+^) and M2 (CD206^+^) macrophages were identified. Representative flow cytometry dot plots of the FACS analysis of F4/80^+^CD86^+^ M1 macrophages (**A**). Percentage of F4/80^+^CD86^+^ M1 macrophages in the lungs (**B**). Representative flow cytometry dot plots of the FACS analysis of F4/80^+^CD206^+^ M2 macrophages (**C**). Percentage of F4/80^+^CD206^+^ M2 macrophages (**D**) in the lungs. The results are presented as the mean ± SEM (*n* = 6). * *p* < 0.05 and *** *p* < 0.001 vs. the ND group, # *p* < 0.05 and ### *p* < 0.001, vs. the HFD-treated group.

Activated macrophages are divided into two groups, classically activated M1 and alternatively activated M2. We analyzed the types of M1 and M2 macrophages in the lungs, because we observed numerous macrophages in BALF. We measured the populations of pro-inflammatory F4/80^+^CD86^+^ M1 and anti-inflammatory F4/80^+^CD206^+^ M2 macrophages in the lungs using flow cytometry. As shown in Figure 4A, the HFD increased the population of M1 macrophages, whereas CID16020046 significantly suppressed the M1 macrophage population. In contrast, the HFD decreased the population of M2 macrophages, and CID16020046 significantly reversed the decrease (Figure 4B).

### 2.5. CID16020046 Suppresses Inflammatory Cytokine Levels in the Lungs

**Figure 5:**
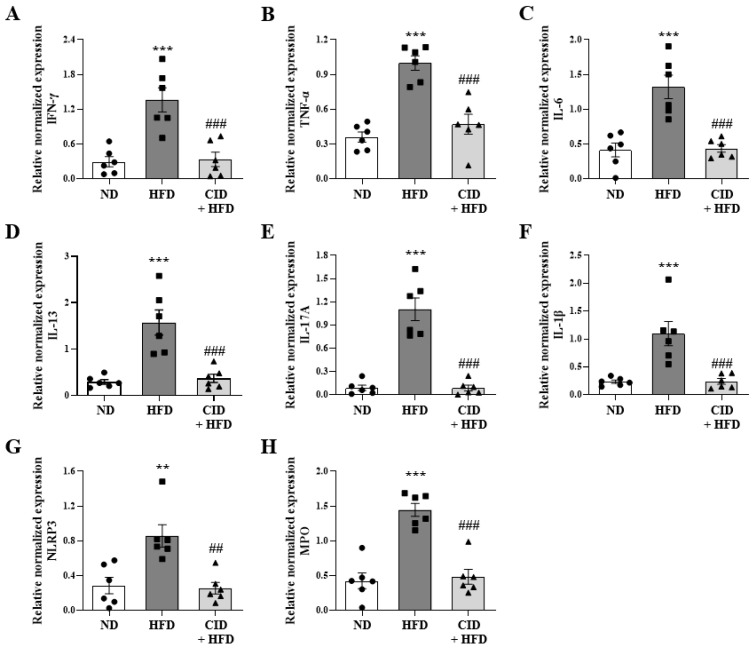
CID16020046 inhibits high-fat diet (HFD)-induced pro-inflammatory responses in the lungs. (**A**–**H**) mRNA expression of *Ifn-γ* (**A**), *Tnf-α* (**B**), *Il-6* (**C**), *Il-13* (**D**), *Il-17a* (**E**), *Il-1β* (**F**), *Nlrp-3* (**G**), and *Mpo* (**H**) in the lungs. The mRNA levels of cytokines were quantified as ratios to mRNA levels of *glyceraldehyde 3-phosphate dehydrogenase* (*Gadph*). The results are presented as the mean ± SEM (*n* = 6). ** *p* < 0.01 and *** *p* < 0.001 vs. the ND group, ## *p* < 0.01 and ### *p* < 0.001, vs. the HFD-treated group.

Obesity is associated with a chronic low-grade pro-inflammatory state, and high expression of several inflammatory mediators such as IL-6 and TNF-α have been implicated [32,33]. The pro-inflammatory gene levels of *Ifn-γ*, *Tnf-α*, *Il-6*, *Il-13*, *Il-17A*, *Il-1β*, *Nlrp3*, and *Mpo* in the lungs were determined by qRT-PCR and were significantly increased in the HFD group (Figure 5A–H), while CID16020046 significantly inhibited these increases (Figure 5A–H). Obesity-associated airway inflammation is facilitated by IL-17-producing type 3 innate lymphoid cells and IL-1β-producing NLRP3 inflammasome [33,34]. Thus, the increased gene expression levels of *Il-17A* and inflammasome-related *Nlrp-3* and *Il-1β* supported the obesity model, and CID16020046 treatment inhibited this increase in the HFD group. (Figure 5E–G). The Th1 genes *Ifn-γ*, *Tnf-α*, and *Il-6*; Th2 gene *Il-13*; and neutrophil marker gene *Mpo* were also significantly increased in the HFD group, while CID16020046 significantly inhibited this increase (Figure 5A–D,H).

### 2.6. CID16020046 Suppressed Inflammatory Cytokine Levels in the Gonadal White Adipose Tissue

**Figure 6:**
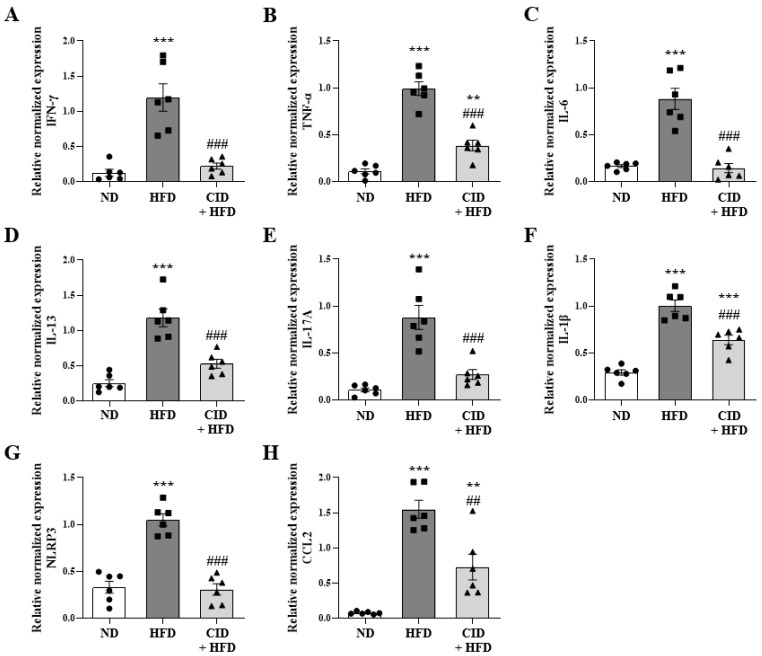
CID16020046 inhibits high-fat diet (HFD)-induced pro-inflammatory responses in gonadal adipose tissue. (**A**–**H**) mRNA expression of *Ifn-γ* (**A**), *Tnf-α* (**B**), *Il-6* (**C**), *Il-13* (**D**), *Il-17a* (**E**), *Il-1β* (**F**), *Nlrp-3* (**G**), and *Ccl2* (**H**) in the gonadal white adipose tissue. The mRNA levels of cytokines were quantified as ratios to mRNA levels of *glyceraldehyde 3-phosphate dehydrogenase* (*Gadph*). The results are presented as the mean ± SEM (*n* = 6). ** *p* < 0.01, *** *p* < 0.001 vs. the ND group, ## *p* < 0.01 and ### *p* < 0.001, vs. the HFD-treated group.

To verify the mechanism by which chronic low-grade systemic inflammation caused by white adipose tissue could contribute to airway inflammation in obese airway inflammation [31], we analyzed the inflammatory cytokine levels by qRT-PCR in the gonadal white adipose tissue. The levels of *Ifn-γ*, *Tnf-α*, *Il-6*, *Il-13*, *Il-17A*, *Il-1β*, *Nlrp3*, and *Ccl-2* genes were significantly increased in the HFD group (Figure 6), while CID16020046 significantly inhibited this increase (Figure 6).

### 2.7. CID16020046 Suppressed Inflammatory Cytokine Levels in the BALF

**Figure 7:**
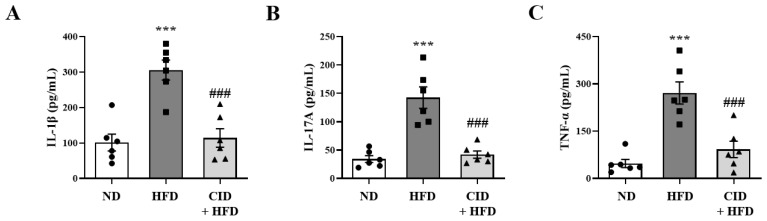
CID16020046 suppresses the high-fat diet (HFD)-induced increase in pro-inflammatory cytokines in bronchoalveolar lavage fluid (BALF). Protein expression of IL-1β (**A**), IL-17A (**B**), and TNF-α (**C**) in BALFs from mice. Protein levels were quantified using enzyme-linked immunosorbent assay. Values represent the means ± SEMs (*n* = 6) *** *p* < 0.001 vs. the ND group, ### *p* < 0.001, vs. the HFD-treated group.

In order to confirm the changes of pro-inflammatory cytokine levels at the protein level, we measured the protein levels of IL-1β, IL-17A, and TNF-α in the BALF. The HFD induced increases in pro-inflammatory genes in BALF (Figure 7A–C). CID16020046 significantly suppressed these increases (Figure 7A–C).

### 2.8. CID16020046 Increases Adiponectin Levels and Suppresses Leptin Levels in the Lungs and Gonadal White Adipose Tissue

**Figure 8:**
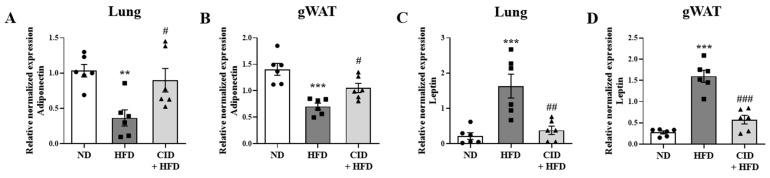
CID16020046 reverses high fat diet (HFD)-induced changes of expression levels of *AdipoQ* and *Lep* in gonadal adipose tissue. (**A**–**D**) mRNA expressions of *AdipoQ* (**A**,**B**) and *Lep* (**C**,**D**) in the lungs (**A**,**C**) and gonadal white adipose tissue (**B**,**D**) of mice. Adipokine mRNA levels were quantified as ratios relative to mRNA levels of *Gapdh*. Values represent the means ± SEMs (*n* = 6). ** *p* < 0.01 and *** *p* < 0.001 vs. the ND group, # *p* < 0.05, ## *p* < 0.01, and ### *p* < 0.001, vs. the HFD-treated group.

Obesity regulates the levels of adipokines, such as adiponectin and leptin [35,36]. We analyzed the levels of *AdipoQ* and *Lep* in the lungs and gonadal white adipose using qRT-PCR. The levels of *AdipoQ* were significantly decreased in both samples of the HFD group, whereas CID16020046 significantly reversed these decreases (Figure 8A,B). The levels of *Lep* increased significantly in the adipose tissue and lungs of HFD group mice, whereas CID16020046 significantly reversed this increase (Figure 8C,D).

### 2.9. Changes of Gut Microbiota Composition by High-Fat Diet (HFD) and CID16020046

**Figure 9:**
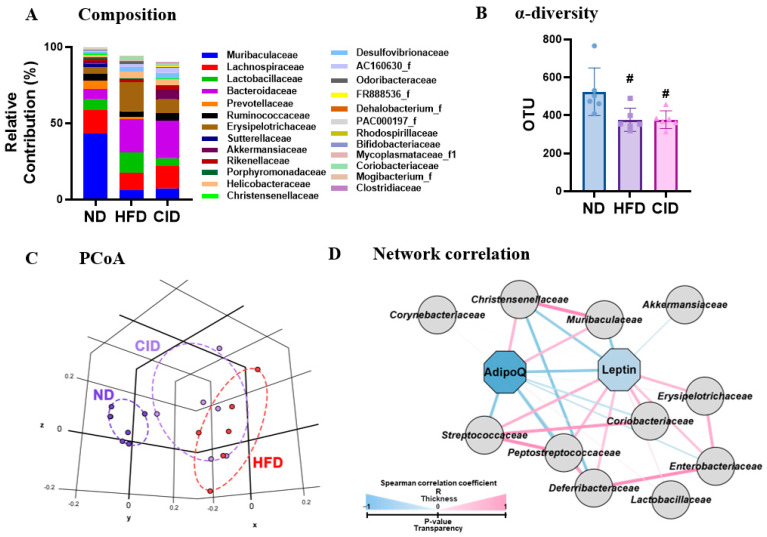
Changes of gut microbiota by high-fat diet (HFD) and CID16020046. (**A**) Composition of gut microbiota at the phylum level. (**B**) α-diversity. (**C**) PCoA. (**D**) Network correlation. Results were obtained from six mice per group. # *p* < 0.05 vs. the ND-treated group.

Given that gut microbiota contributes to obesity, we performed meta-genome analysis of the feces from mice to understand whether the HFD and CID16020046 affect gut microbiota composition. The HFD feeding showed a significant decrease in *Bacteroidetes* (*Bacteriodota*) and *Verrucomicrobia* populations and an increase in *Firmicutes* (*Bacillota*) and *Actinobacteria* (*Actinomycetota*) populations at the phylum level, which reproduces the patterns in obese human and rodents [37,38]. At the family level, the HFD decreased *Muribaculaceae*, *Lachnospiraceae*, *Prevotellaceae*, *Ruminococcaceae*, *Sutterellaceae*, and *Akkermansiaceae*, with increases of *Lactobacillaceae*, *Bacteroidaceae*, *Erysipelotrichaceae*, *AC160630_f*, *Odoribacteraceae*, and *Coriobacteriaceae* populations (Figure 9A) [37,38]. However, CID16020046 treatment increased HFD-induced populations of *Akkermansiaceae*, *Lachnospiraceae, Muribaculaceae*, and *Christensenellaceae*, while the HFD suppressed populations of *Lactobacillaceae*, *Erysipelotrichaceae*, *Lactobacillaceae*, *Erysipelotrichaceae*, *Odoribacteraceae*, and *Coriobacteriaceae* (Figure 9A), reflecting similar patterns observed in healthy humans and rodents [39,40]. Furthermore, the HFD decreased α-diversity (OUT richness), which indicates the number of species present. CID16020046 treatment did not reverse the decreased α-diversity (Figure 9B). The HFD shifted β-diversity (PCoA), with differences in the overall taxonomic composition that were more dissimilar to the ND group. CID16020046 treatment changed the HFD-shifted β-diversity to more closely resemble the ND group, the change was weak and non-significant (Figure 9C). To analyze the correlation between obesity factors and intestinal microorganisms, we performed a network correlation analysis between intestinal microbiota composition and expression levels of *AdipoQ* and *Lep*. *AdipoQ* levels were positively correlated with *Muribaculaceae* and *Christensenllaceae* populations and negatively correlated with *Lep* expression levels (Figure 9D). In contrast, *AdipoQ* expression levels were negatively correlated with *Streptococcaceae* and *Peptostreptococcaceae* populations, whereas *Lep* levels were positively correlated with *Streptococcaceae*, *Peptostreptococcaceae*, *Coriobactelaceae*, and *Erysipelotrichaceae* populations (Figure 9D).

## 3. Discussion

CID16020046 administration inhibited HFD-induced airway inflammation, including AHR, immune cell accumulation, mucus hypersecretion, and fibrosis in BALF and lungs. Further investigation of pro-inflammatory genes in the lungs and white adipose tissue supports the chronic low-grade inflammatory status in obese mice; the mRNA levels of *Ifn-γ*, *Tnf-α*, *Il-6*, *Il-13*, *Il-17a*, *Nlrp-3*, *Il-1β*, and *Mpo* genes were upregulated by the HFD and reversed by CID16020046. IL-6, TNF-α, and leptin are well-known pro-inflammatory genes upregulated in patients with obesity [31,32]. Upregulation of *Nlrp-3*, *Il-1β*, and *Il-17a* supports the involvement of IL-17 producing type 3 innate lymphoid cells and the Nlrp-3 inflammasome in obesity-induced airway inflammation [33,34]. An increase in the expression of *Ifn-γ* and *Mpo* implies the presence of neutrophils. In combination with the results of immune cell analysis of BALF and pro-inflammatory genes in the lungs, high numbers of macrophages and neutrophils in BALF were deeply involved in HFD-induced obese airway inflammation. Considering the majority of macrophage population in the BALF compared to the minority of neutrophil population, phenotypes of macrophages in the lungs may strongly influence the status of obese airway inflammation. The HFD increased the pro-inflammatory M1 macrophage portion and decreased the anti-inflammatory M2 macrophage portion, whereas CID16020046 administration reversed the changes. This switching of macrophage phenotypes from M1 to M2 may play an important role in the therapeutic efficacy of CID1602046 in obesity-induced airway inflammation. Furthermore, the increase in pro-inflammatory genes in the lungs may be caused by chronic low-grade inflammation in white adipose tissue, because the changes are stronger in adipose tissue than in the lungs [31,32]. Anti-inflammatory effects of GPR55 antagonists have also been observed in other animal models. CID16020046 administration can reduce experimental intestinal inflammation through GPR55 by reducing macrophage migration and activation [41]. In a dextran sodium sulfate model of colitis, levels of TNF-α and IL-1β were reduced in colon tissues by the daily application of CID16020046 [41]. *Gpr55* gene-deficient mice showed reduced inflammation scores as compared to wild type mice in this model [41]. The anti-neuroinflammatory effect of KIT 10, another GPR55 antagonist, was observed in lipopolysaccharide-activated primary microglial cells via potent inhibition of lipopolysaccharide-induced synthesis of prostaglandin E_2_ [42]. In db/db mice, ML-193, another GPR55 antagonist, suppressed the plasma TNF-α and IL-6 levels, and expression of IL-6, monocyte chemoattractant protein-1, and IL-1β in the liver and epididymal fat [15]. O-1602 activation of GPR55 increases pro-inflammatory cytokines in monocytes and natural killer cells [13].

Results from *Gpr55* gene-deficient mice have been inconsistent. Increased food intake, increased fat mass, and decreased physical activity have been reported in *Gpr55* gene-deficient mice [21,27,43,44]. Another study described subtle differences in food intake and motor activity [45]. However, in the present study, we found no change in food intake after CID16020046 administration. We also observed that CID16020046 administration improved glucose intolerance [10,22,26]. In several in vivo studies, the GPR55 agonist O-1602 improved glucose tolerance and increased glucose-stimulated insulin secretion [3,9], whereas Meadow et al. showed normal glucose tolerance with no differences in GSIS in *Gpr55* gene-deficient mice [44]. Our data suggest that CID16020046 blockage of GPR55 leads to improved glucose intolerance. Since the effects of O-1602 were observed in *Gpr55* gene-deficient mice, the effects of O-1602 should be interpreted carefully [46]. Although further investigation is necessary to clarify the functions of GPR55 in glucose homeostasis in *Gpr55* gene-deficient mice, our data suggest that CID16020046 blockage of GPR55 might be a therapeutic option for obesity-induced airway inflammation.

## 4. Materials and Methods

### 4.1. Materials

CID16020046 was purchased from Tocris Bioscience (Bristol, UK). A rodent diet containing 60 kcal% fat (cat. D12492) was purchased from Research Diets (New Brunswick, NJ, USA). All other chemicals were purchased from Sigma-Aldrich (St. Louis, MO, USA).

### 4.2. Mouse Strain

All experimental protocols involving live mice were approved by the Institutional Animal Care Committee of Kyung Hee University (KHSASP-23-019). Daehan Biolink (DBL, Seoul, Republic of Korea) provided 5-week-old male C57BL/6 mice, which housed two mice per cage in a room maintained at 22–24 °C and a relative humidity of 60 ± 5%. Standard laboratory chow and water were provided ad libitum.

### 4.3. HFD Feeding

Male C57BL/6 mice obtained from DBL had ad libitum access to water and food at the laboratory animal facility of Kyung Hee University. Six-week-old mice were randomly divided into three groups. Control C57BL/6 mice (n = 6) were fed a normal chow diet (ND; DBL) for 15 weeks. HFD C57BL/6 mice (n = 6) were fed a synthetic diet supplemented with 60% (*w*/*w*) as the HFD (Research Diets, New Brunswick, NJ, USA) for 15 weeks (Figure 1A). Mice in the HFD plus CID16020046 group were treated with CID16020046 (1 mg/kg) by intraperitoneal injection five days per week for the last 5 weeks (n = 6) (Figure 1A).

### 4.4. Oral Glucose Tolerance Testing (OGTT)

For the OGTT, the mice were fasted for 6 h before the experiment. D-(+)-Glucose was administered orally at 2 g/kg body weight. Glucose levels in the tail blood were measured using BAROzen II glucometers and strips (Handok, Seoul, Republic of Korea) at 0, 15, 30, 60, and 120 min.

### 4.5. Measuring Airway Hyperresponsiveness (AHR) to Methacholine

AHR was evaluated using a noninvasive lung function measurement Model PLY-UNR-MS2 (EMKA Technologies, Paris, France). After placing the mice in a barometric plethysmographic chamber, the baseline was recorded for 3 min and the enhanced pause (Penh) was calculated according to the manufacturer’s protocol. The results are expressed as the percentage increase in Penh following challenge with increasing concentrations of methacholine (0, 6.25, 12.5, 25, and 50 mg/mL)

### 4.6. Bronchial Alveolar Lavage Fluid (BALF) Cell Enumeration and Analysis

The immune cells in the BALF were collected in 0.4 mL PBS. Twenty microliters of this sample was mixed with 20 μL of Wright’s solution. The 40 μL volume was loaded into the hemocytometer. After counting the number of cells, the total number of cells was calculated by multiplication. For the May–Grünwald–Giemsa staining, BALF cells in 50 μL of cell suspension were adhered to a glass slide using a CellSpin^®^ centrifuge (Hanil Electric, Seoul, Republic of Korea), fixed in methanol for 30 s, and stained with May–Grünwald solution for 8 min, followed by Giemsa solution for 12 min. Based on the cellular staining and morphological characteristics, the cells were classified as macrophages, lymphocytes, or neutrophils. In each case, 500 cells were counted and the percentage of each cell type was calculated.

### 4.7. Histological Examination of Lung Tissue

Lung tissue sections prepared from the lungs were stained with hematoxylin and eosin (H&E), periodic acid-Schiff (PAS), and Masson’s trichrome (MT) to evaluate immune cell infiltration, mucus-producing cells, and fibrosis, respectively [47].

### 4.8. Quantitative Real-Time PCR (qRT-PCR)

Total RNA in the lungs and gonadal white adipose tissue was isolated using TRIzol^®^ (Invitrogen, Waltham, MA, USA). The RNA was reverse transcribed into cDNA using MMLV reverse transcriptase (Promega, Madison, WI, USA). Thunderbird Next SYBR qPCR Mix was used for qRT-PCR on a CFX Connect Real-Time System (Bio-Rad, Hercules, CA, USA). Specific primers of *Ifn-γ* (sense 5′-CGCTACACACTGCATCTTGG-3′, antisense 5′-TTTCAATGACTGTGCCGTGG-3′), *Tnf-α* (sense 5′-ACGGCATGGATCTCAAAGAC-3′, antisense 5′-AGATAGCAAATCGGCTGACG-3′), *Il-6* (sense 5′-CTGATGCTGGTGACAACCAC-3′, antisense 5′-TCCACGATTACCCAGAGAAC-3′), *Il-13* (sense 5′-CAGCATGGTATGGAGTGTGG-3′, antisense 5′-AGGCCATGCAATATCCTCTG-3′), *Il-17A* (sense 5′-AAAGCTCAGCGTGTCCAAAC-3′, antisense 5′-ACGTGGAACGGTTGAGGTAG-3′), *Nlrp-3* (sense 5′-ATGCTGCTTCGACATCTCCT-3′, antisense 5′-GTTTCTGGAGGTTGCAGAGC-3′), *Il-1β* (sense 5′-CAGGCAGGCAGTATCACTCA-3′, antisense 5′-TGTCCTCATCCTGGAAGGTC-3′), *Mpo* (sense 5′-CACTGGACACTGCAACAACA-3′, antisense 5′-CCATTGCGATTGACTCCAGG-3′), *Ccl2* (sense 5′-TGAATGTGAAGTTGACCCGT-3′, antisense 5′-ACAGAAGTGCTTGAGGTGGT-3′), *Lep* (sense 5′-TTCCTGTGGCTTTGGTCCTA-3′, antisense 5′-CGACTGCGTGTGTGAAATGT-3′), *AdipoQ* (sense 5′-TACTGCAACATTCCGGGACT-3′, antisense 5′-GTAGGTGAAGAG AACGGCCT-3′), and *Gapdh* (sense 5′-AACTTTGGCATTGTGGAAGG-3′, antisense 5′-GGATGCAGGGATGATGTTCT-3′) were used.

### 4.9. Flow Cytometry

For the identification of M1/M2 macrophages from mouse lung tissue, single-cell suspensions were prepared using collagenase type II (cat. 17101015, Gibco, Grand Island, NY, USA). For M1/M2 macrophage analysis, single cells prepared from the lungs were stained with an FITC-labeled rat antibody against F4/80 (cat. 11-4801-81; eBioscience, San Diego, CA, USA) and an APC-labeled rat antibody anti-CD86 (cat. 17-0862-81; eBioscience) or anti-CD206 (cat. 17-2061-82; eBioscience) at 4 °C for 45 min. After fixing with IC fixation buffer (cat. 00-8222-49; eBioscience) at 22 °C for 1 h, a CytoFLEX Flow cytometer (Beckman Coulter, Brea, CA, USA) was used for cell analysis.

### 4.10. Enzyme-Linked Immunosorbent Assay (ELISA)

Interleukin (IL-1β), IL-17A, and tumor necrosis factor-alpha (TNF-α) levels in the BALF of mice were quantified using ELISA kits (eBioscience, San Diego, CA, USA). Capture and biotinylated detection antibodies specific for IL-1β (cat. BMS6002TWO) and IL-17A (cat. No. 88-7371-88), and TNF-α (cat. 88-7324-88) were obtained from eBioscience. Avidin-horseradish peroxidase (HRP) was used, and absorbance was measured at 450 nm.

### 4.11. Analysis of Gut Microbiota

Bacterial genomic DNA was extracted from fresh mouse feces using a QIAamp DNA Stool Mini Kit. Extracted genomic DNA was amplified using V4-barcoded primers targeting the bacterial 16S rRNA gene. Each amplicon was sequenced using an iSeq 100 system (Illumina, San Diego, CA, USA). The extracted FASTQ data were processed and analyzed using the EzBioCloud pipeline (CJ Bioscience, Seoul, Republic of Korea).

In the network analysis, correlation analysis was performed focusing on classification units with high correlation and statistical significance at the family level. This selection was based on the Spearman correlation coefficient applied to both the processed analytical data and behavioral experimental data obtained from all datasets. For visualization, Cytoscape was used to show the positive correlations in pink and negative correlations in blue. The thickness of the line represents the strength of the correlation and the transparency of the line represents the *p*-value.

### 4.12. Statistics

All data are presented as mean ± SEM (*n* = 6). One-way ANOVA was used to evaluate the treatment effect, followed by Tukey’s multiple comparison test. Statistical significance was analyzed using GraphPad Prism version 5 (GraphPad, San Diego, CA, USA). Statistical significance was set at *p* < 0.05. * *p* < 0.05, ** *p* < 0.01, and *** *p* < 0.001 vs. the ND group, # *p* < 0.05, ## *p* < 0.01, and ### *p* < 0.001, vs. the HFD-treated group.

## Data Availability

Data are available from the authors upon request.
